# Efficacy of endometrial microbiota testing-guided personalized therapy in infertile women stratified by CD138 status: a retrospective cohort study

**DOI:** 10.3389/fendo.2026.1849095

**Published:** 2026-08-26

**Authors:** Fang He, E’Nuo Peng, Jiao Lin, Zhuo Mao, Xiaowei Lu, Hui Xu, Lei Guo, Yangyun Zou, Jing Wang, Sijia Lu, Xianghong Huang

**Affiliations:** 1 Center for Reproduction and Genetics, Xiangtan Central Hospital, Xiangtan, Hunan, China; 2 The Third Xiangya Hospital of Central South University, Changsha, Hunan, China; 3 Yikon Genomics Co., Ltd., Shanghai, China

**Keywords:** 16S rRNA, intrauterine microbiome, IVF, personalized treatment, pregnancy outcomes

## Abstract

**Background:**

Chronic endometritis (CE) is a persistent inflammatory condition of the endometrium associated with infertility. Current diagnosis relies on histopathological markers like CD138, which may not fully assess the functional state of the endometrial microenvironment. This study aimed to investigate whether the endometrial microbiome test (EMT) combined with personalized therapy could improve pregnancy outcomes in infertile women undergoing IVF, compared with standard management based on CD138 results.

**Methods:**

This retrospective cohort study included 336 infertile women (aged 22–37 years) who underwent the CD138 test in Xiangtan Central Hospital between January 2020 and December 2022. Among them, 162 patients opted to undergo concurrent EMT using 16S rRNA sequencing and received EMT-guided personalized treatment. After excluding patients with missing CD138 results, those who underwent sequential embryo transfer, and those with endometrial thickness <7 mm at the time of transfer, 151 patients were included in the EMT group and 155 in the non-EMT group. The non-EMT group received standard management based on CD138 results (empirical antibiotics for CD138-positive patients; no treatment for CD138-negative patients). Clinical outcomes were compared between the two groups overall and after stratification by CD138 status. Multivariable logistic regression was performed to adjust for confounders.

**Results:**

The overall live birth rate in the EMT group was 64.24% (97/151), which was higher than the non-EMT group (46.45%, 72/155, adjusted p=0.004). Among CD138-negative patients, EMT-guided therapy was associated with a significantly higher clinical pregnancy rate (70.89% vs. 53.41%, adjusted p=0.011), ongoing pregnancy rate (62.03% vs 46.59%, adjusted p=0.041), and live birth rate (60.76% vs. 43.18%, adjusted p=0.030) compared to no treatment. Among CD138-positive patients, EMT-guided therapy showed no significant improvement for the ongoing pregnancy rate (69.44% vs. 52.24%, adjusted p=0.142) or the live birth rate (68.06% vs. 50.75%, adjusted p=0.118) compared to empirical antibiotic treatment.

**Conclusion:**

EMT-guided personalized therapy was associated with favorable reproductive outcomes in CD138-negative women with infertility, identifying a subgroup that may benefit from microbiota-targeted intervention. For CD138-positive patients, the added value of EMT over empirical antibiotic therapy remains uncertain and warrants further investigation. EMT may guide treatment in CD138-negative cases via sequential diagnosis, though prospective validation is needed.

## 1 Introduction

*In vitro* fertilization (IVF) and embryo transfer (ET) have emerged as fundamental components of assisted reproductive technology (ART), providing a critical treatment pathway for millions of infertile couples. However, the success of IVF remains a significant challenge, with clinical pregnancy rates of around 30–40% per cycle (1). This has driven researchers to explore novel factors that may influence reproductive outcomes, particularly those related to the uterine environment. Chronic endometritis (CE) is an important uterine factor of infertility and is strongly associated with recurrent implantation failure and recurrent pregnancy loss (2–4). CE is generally diagnosed based on hysteroscopic findings and the endometrial CD138 test (5, 6); however, the diagnostic accuracy of these methods remains controversial due to a lack of standardized criteria. Discrepancies in biopsy timing, sampling techniques, diagnostic markers, and interpretation can lead to both underdiagnosis and overdiagnosis. This diagnostic inconsistency also presents a significant challenge for drawing reliable conclusions from research on the effectiveness of various CE treatments (7–9).

The intrauterine microbiome, though less diverse than gut or vaginal microbiota, plays a critical role in maintaining uterine health and facilitating successful embryo implantation (10). Dysbiosis within this microbial community has been linked to adverse reproductive outcomes, including implantation failure, recurrent pregnancy loss, and preterm birth (11, 12). A Lactobacillus-dominated intrauterine microbiome has been associated with higher implantation and pregnancy rates, while the presence of pathogenic bacteria such as Gardnerella or Atopobium correlates with poorer reproductive outcomes (13, 14).

The emergence of high-throughput sequencing technologies, particularly 16S ribosomal RNA (rRNA) gene sequencing, now allows detailed characterization of the intrauterine microbiome (15). Recent studies have demonstrated that 16S rRNA-based analysis can identify patients at risk of IVF failure and guide targeted interventions to restore microbial balance (16, 17). In IVF, personalized approaches that address the unique microbial profile of the intrauterine environment may offer a promising strategy to improve pregnancy outcomes. For instance, targeted antibiotic therapy, probiotics, or immunomodulatory agents can be used to correct dysbiosis and create a more favorable environment for embryo implantation (18–20).

Despite these advances, comparative studies evaluating the impact of microbiome-based personalized treatment on IVF outcomes are still lacking. In particular, it remains unclear whether EMT-guided therapy offers additional benefit over standard management based on CD138 results alone, and whether its efficacy differs according to CD138 status. This study aimed to investigate whether EMT-guided personalized therapy improves pregnancy outcomes in infertile women compared with standard management, and to explore whether the treatment effect differs between CD138-positive and CD138-negative patients.

## 2 Methods

### 2.1 Study design and population

This retrospective cohort study evaluated the clinical outcomes of infertile women undergoing IVF treatment at Xiangtan Central Hospital between January 2020 and December 2022, comparing those who received EMT-guided personalized therapy (EMT group) with those who received standard management based on CD138 results alone (non-EMT group). Infertile women underwent CD138 testing for the following indications: previous failures of embryo transfer, recurrent pregnancy loss (RPL), or clinical suspicion of chronic endometritis based on sonographic findings (e.g., endometrial polyps, fluid accumulation, or intrauterine adhesions). Patients indicated for CD138 testing were counseled regarding the option of concurrent EMT. Those who opted to undergo concurrent EMT testing provided written informed consent and agreed to receive personalized treatment based on their EMT results. All patients in both groups underwent CD138 testing, and the results were used for stratification analysis.

**Figure 1:**
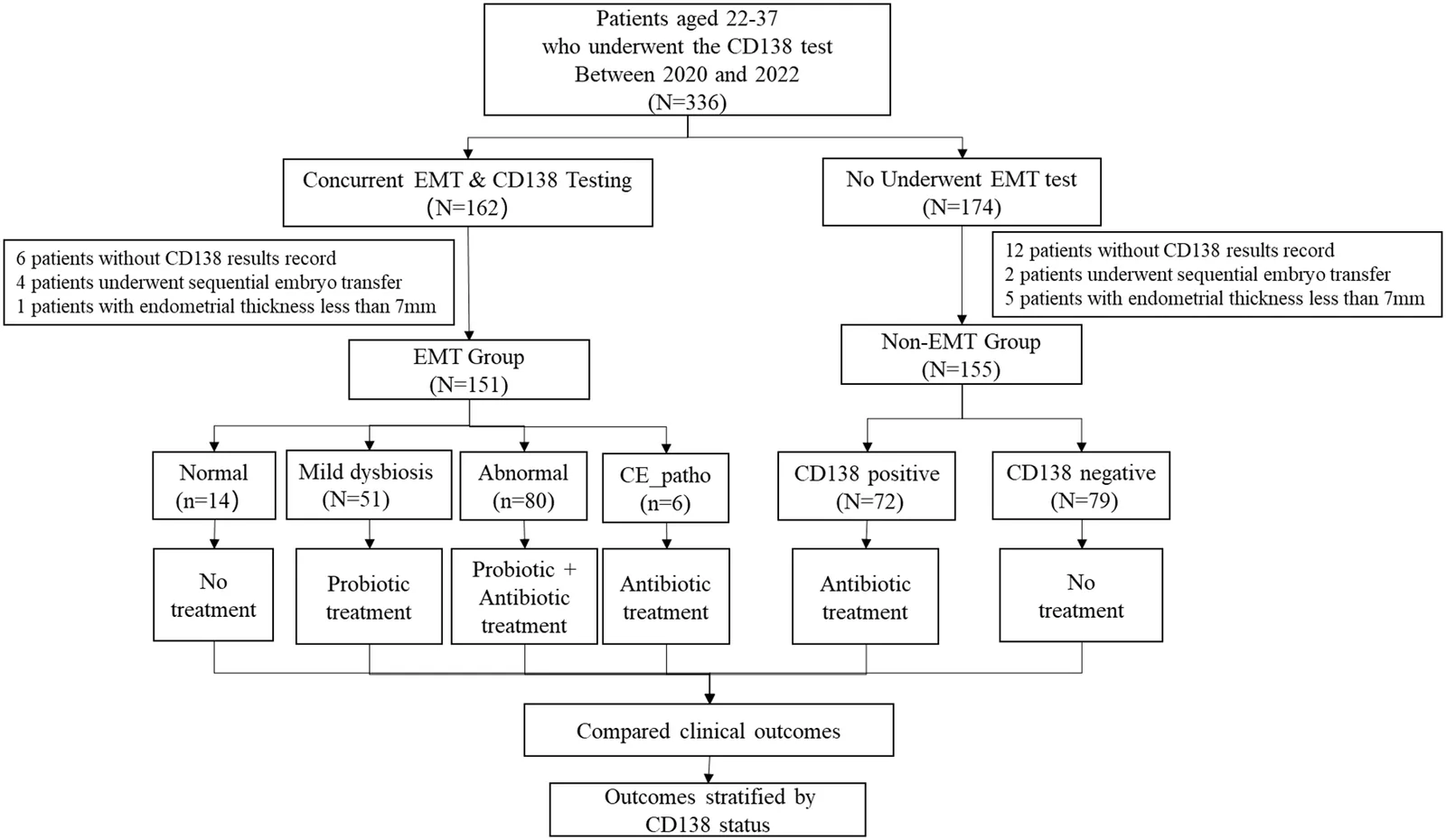
The flowchart of research design. Normal: with normal endometrial microbiota composition, did not test positive for the pathogenic bacteria causing chronic endometritis; Mild dysbiosis: with abnormal endometrial microbiota composition, did not test positive for the pathogenic bacteria causing chronic endometritis; Abnormal: with abnormal endometrial microbiota composition tested positive for the pathogenic bacteria causing chronic endometritis; CE_patho: with normal endometrial microbiota composition, tested positive for the pathogenic bacteria causing chronic endometritis.

To minimize the influence of embryonic factors, we included only women aged 22–37 years who underwent cleavage-stage or blastocyst embryo transfer. The same exclusion criteria were applied to both groups: without CD138 results, undergoing sequential embryo transfer, and endometrial thickness less than 7mm at the time of transfer. Ultimately, 151 patients were included in the EMT group and 155 in the non-EMT group (**Figure 1**).

The study was conducted in accordance with the ethical principles outlined in the Declaration of Helsinki and was approved by the ethics committee of Xiangtan Central Hospital (Approval ID: S2-202206002). All patients gave their written consent for the anonymous use of their clinical data for research purposes.

### 2.2 Sample collection process

The endometrial tissue was sampled on the day of LH + 7 (natural cycle), or P + 5 (artificial hormone cycle) using a disposable sterile endometrium sampler (Yikon Inc.). The sampler is designed with an outer layer that shields its inner tube from contamination by the vagina and cervix. Briefly, with the patient in the lithotomy position, the vulva was disinfected, and after introduction of the disinfected speculum, the external cervical os was cleaned with a dry swab to remove mucus or debris. Then the sampler was gently inserted towards the direction of the uterus, and the endometrial tissue was obtained by suction. To avoid contamination, the catheters were kept clear of the vaginal wall. After collection, their outer surfaces were wiped with sterile gauze, and their contents were placed into cryotubes prefilled with 1.5 mL of RNAlater solution (XK-039, Yikon Inc.). All samples were stored at – 80°C until use.

### 2.3 Immunohistochemical analysis of CD138

The standardized protocol of CD138 IHC comprised: 1) mounting 4-μm formalin-fixed paraffin-embedded (FFPE) endometrial sections on poly-L-lysine-coated slides; 2) deparaffinization in xylene and rehydration through graded alcohols; 3) heat-mediated antigen retrieval in EDTA buffer (pH 9.0, 95 °C, 20 min); 4) blocking endogenous peroxidase with 3% H_2_O_2_; 5) overnight incubation at 4 °C with monoclonal mouse anti-CD138 primary antibody (Clone MI15, Dako, 1:100); 6) application of HRP-conjugated secondary antibody; 7) signal amplification using StreptAvidin-Biotin Complex (SABC); 8) DAB chromogen development; 9) hematoxylin counterstaining; 10) dehydration and mounting.

CD138+ endometrial stromal plasma cells (ESPCs) exhibited strong membranous positivity with weak cytoplasmic staining (distinct brown DAB deposition), characterized by eccentric round nuclei, coarse chromatin arranged in a radial “cartwheel” pattern, and basophilic cytoplasm. Chronic endometritis was diagnosed when ≥1 typical CD138+ ESPCs were identified per 400× high-power field (HPF; field diameter 0.55 mm).

### 2.4 RNA extraction and sequencing

Total RNA from endometrial samples, including human and microbial RNA, was extracted using the RaPure Total RNA Micro Kit (Catalog Number: R4012, Magen Biotech, China). Quantification of RNA was performed using a Qubit 4 Fluorometer and RNA HS kit (Catalog Number: Q32852, ThermoFisher, USA). Reverse transcription, V-region amplification, and Next Generation Sequencing (NGS) library preparation were carried out according to the manufacturer’s instructions using the EMT library kit (Catalog Number: KT100807048, Yikon Genomics, China). SE400 sequencing was conducted on an Ion Torrent S5 sequencer (ThermoFisher, USA), with each library yielding 1M raw reads. To establish a blank reference baseline, we prepared and sequenced 20 blank control samples (ultrapure water) using the same reagent batch and protocol.

### 2.5 Endometrium microbiota analysis

The obtained reads from the 16S rRNA sequencing were preprocessed using fastp software (v0.20.1). Then the processed reads were trimmed according to the primer sequences of six variable regions using cutadapt (v3.4). To ensure data quality, samples with >50,000 quality-filtered reads were retained for downstream analysis. Denoising and inference of amplicon sequence variants (ASVs) were performed using the DADA2 method within QIIME 2 (v2021.8). Potential contaminant sequences were identified and removed using the decontam algorithm (prevalence method) via the QIIME2 decontam-remove function (21). Taxonomic assignment of ASVs was conducted with the vsearch classifier against the Silva database (v1.32). Finally, relative abundances of microbial taxa were calculated based on ASV counts in each biopsy sample.

CE-associated pathogens were defined based on established literature and clinical consensus (22–27). The following microorganisms were considered pathogenic for chronic endometritis (CE): *Escherichia* spp.*, Klebsiella* spp.*, Shigella* spp.*, Neisseria* spp.*, Enterococcus* spp.*, Staphylococcus* spp.*, Streptococcus* spp.*, Chlamydia trachomatis, Mycoplasma, Ureaplasma, and Mycobacterium tuberculosis*. Endometrial samples positive for any of these pathogens were classified as CE pathogen-positive. A sample was considered positive for a given pathogen when its relative abundance exceeded 0.1% of the total bacterial sequences, to minimize the impact of potential low-abundance contamination (28, 29).

*Lactobacillus*-dominated microbiota was defined as having a relative abundance of *Lactobacillus* spp. ≥ 50%. The 50% threshold was empirically determined based on the bimodal distribution of *Lactobacillus* relative abundance in our internal cohort of 1,000 clinical samples (median: ~48%), which included both healthy women and patients with recurrent implantation failure (unpublished data). As no universal consensus exists for defining *Lactobacillus*-dominated endometrial microbiota, this data-driven cut-off was selected to optimally stratify the samples into two distinct microbiota types.

The results of EMT can be divided into four subgroups. 1) Normal subgroup: the dominant *Lactobacillus* with proportion over 50% and related-pathogens not detected; 2) Abnormal subgroup: the proportion *Lactobacillus* is less than 50% and Chronic Endometritis (CE) pathogens are detected; 3) Mild dysbiosis subgroup: the proportion *Lactobacillus* is less than 50% and CE pathogens are not detected; 4) CE pathogens subgroup: the dominant *Lactobacillus* with proportion over 50% and CE pathogens are detected.

### 2.6 Antibiotic treatment

Patients in the EMT group received personalized treatment based on their microbiomic profiles: The normal subgroup did not require intervention. The Mild dysbiosis subgroup was treated with intravaginal Lactobacillus suppositories for 10 days and oral probiotics. The product used is a live Lactobacillus suppository approved by China’s National Medical Products Administration (NMPA), containing *Lactobacillus delbrueckii* DM8909 as the active ingredient (30). The abnormal subgroup: received pathogen-directed antibiotic therapy followed by probiotic supplementation. The CE pathogens subgroup: received targeted antibiotics without probiotics. The recommended antibiotic and probiotic regimens for each pathogen and microbiota profile are summarized in **Supplementary Table 1**.

The control group of CD138-positive patients was treated according to the empirical antibiotic treatment (Doxycycline, for 14 days, or Ciprofloxacin, plus Metronidazole, for 14 days), and no treatment was administered to CD138-negative patients. All CD138-positive patients completed their assigned antibiotic course and proceeded to embryo transfer in a subsequent menstrual cycle. Both groups were managed under the same follow-up protocol.

### 2.7 Controlled ovarian stimulation and frozen embryo transfer

Controlled ovarian stimulation was conducted following our center’s standard protocols, including the GnRH agonist long protocol, GnRH antagonist protocol, or mild stimulation approaches. Fertilization was achieved via conventional IVF or ICSI, with subsequent embryo vitrification performed on either Day 3 or Day 5/6 of culture. Before initiating the frozen embryo transfer cycle, we would confirm whether the patient has completed the course of antibiotic treatment. The hormone replacement therapy (HRT) cycle protocol was initiated on menstrual cycle days 2–3. Patients received oral estradiol valerate (Bayer AG, Germany) at 3 mg twice daily for 7 days. Estrogen dosage was subsequently adjusted based on endometrial thickness monitoring. When the endometrium reached ≥8 mm, progesterone was administered for endometrial transformation. Cleavage-stage embryos (day-3) were transferred on the third day post-progesterone initiation, while blastocysts were transferred on the fifth day. The embryos were thawed using the commercial thawing kit (Kitazato, Japan), and after thawing, the embryos were transferred according to the clinical routine.

### 2.8 Statistical analysis

Categorical variables were compared using the Pearson χ2 test or Fisher’s exact test. Continuous variables were analyzed using the t-test when normality held and the Wilcoxon rank-sum test otherwise. Multiple logistic regression analyses were conducted to compare the outcomes of the EMT group and the control groups after controlling for covariates with *P <* 0.20 and covariates associated with IVF outcomes, including female age, female BMI, basal Follicle-Stimulating Hormone (FSH), AMH, duration of infertility, previous IVF failures, number of prior miscarriages, IVF indication, type of infertility, endometrial thickness, number of embryos transferred, and grade/stage of embryo transfer.

Statistical analyses were performed using the R language (Version 4.3.3). A p-value < 0.05 was considered statistically significant.

## 3 Results

### 3.1 Study population and EMT subgroup classification

A total of 151 patients in the EMT group and 155 patients in the non-EMT group were included in the final analysis. Based on their EMT profiles, the 151 EMT participants were stratified into four subgroups: Normal (n = 14), Mild Dysbiosis (n = 51), Abnormal Dysbiosis (n = 80), and CE pathogens (n = 6). As shown in **Table 1**, Endometrial *lactobacilli* deficiency was highly prevalent in the cohort, affecting 86.8% (131/151) of participants, and 57.0% (86/151) of patients had detectable pathogenic bacteria. The prevalence of pathogenic bacteria did not differ significantly between CD138-negative and CD138-positive patients (p = 0.247).

**Table 1:** Endometrial microbiota profiles stratified by CD138 status.

Microbiota profile	Total (n = 151)	CD138-Negative (n=79)	CD138-Positive (n=72)	P-value*
Lactobacillus abundance < 50%, n (%)	131 (86.8%)	68 (86.1%)	63 (87.5%)	0.791
EMT classification, n (%)				/
Normal	14 (9.3%)	9 (11.4%)	5 (6.9%)	
Mild dysbiosis	51 (33.8%)	30 (38.0%)	21 (29.2%)	
Abnormal dysbiosis	80 (53.0%)	38 (48.1%)	42 (58.3%)	
CE pathogens	6 (4.0%)	2 (1.3%)	4 (5.6%)	
Any pathogenic bacteria detected†, n (%)	86 (57.0%)	41 (51.9%)	45 (62.5%)	0.247

*Data are n (%). P-value for comparison between CD138-negative and CD138-positive groups (chi-square test), P-values are not shown for rows with small subgroup sizes (e.g., CE pathogens, n = 6) where statistical comparison would be unreliable.

^†^Pathogenic bacteria include: *Ureaplasma, Enterococcus, Staphylococcus, Streptococcus, Klebsiella, Neisseria, Chlamydia, Mycoplasma,* and *Mycobacterium*.

### 3.2 Clinical outcomes by endometrial microbiota subgroup

Following personalized treatment, the overall live birth rate in the EMT group was 64.24%. The clinical outcomes of the four subgroups are presented in **Table 2**. Due to the small sample size of the CE pathogens subgroup (n = 6), statistical comparisons involving this subgroup were not performed.

**Table 2:** Clinical outcomes according to EMT subgroups and corresponding treatment.

Group	Outcomes			Treatment
	Clinical pregnancy rate	Ongoing pregnancy rate	Live birth rate	
**Non-EMT Group (n=155)**	**55.48% (86/155)**	**49.03% (76/155)**	**46.45% (72/155)**	No Treatment or Antibiotic Treatment
**EMT group (n=151)**	**71.52% (108/151)**	**65.56% (99/151)**	**64.24% (97/151)**	
Normal (n=14)	85.71% (12/14)	78.57% (11/14)	71.43% (10/14)	No Treatment
Mild dysbiosis (n=51)	76.47% (39/51)	70.59% (36/51)	68.63% (35/51)	Probiotic Treatment
Abnormal (n=80)	67.5% (54/80)	61.25% (49/80)	61.25% (49/80)	Probiotic Treatment and Antibiotic Treatment
CE_patho (n=6)	50.00% (3/6)	50.00% (3/6)	50.00% (3/6)	Antibiotic Treatment
P value †	**0.005**	**0.006**	**0.004**	

Data are presented as rates (n/N). Bold values indicate the overall comparison between the Non-EMT group and the EMT group and a statistically significant difference (P <; 0.05).

† Adjusted P-values were derived from multivariate logistic regression analysis, controlling for maternal age, female BMI, basal FSH, AMH, endometrial thickness, duration of infertility, previous IVF failures, number of prior miscarriages, IVF indication, type of infertility, number of embryos transferred, and grade/stage of embryo transfer. P < 0.05 was considered statistically significant.

### 3.3 Clinical outcomes: EMT group vs. non-EMT group

Compared to the non-EMT group, which was managed by CD138 results (no treatment for CD138-negative patients and empirical antibiotic therapy for CD138-positive patients), EMT group demonstrated significantly superior outcomes: clinical pregnancy rate (71.52% vs. 55.48%, adjusted p=0.005), ongoing pregnancy rate (65.56% vs. 49.03%, adjusted p=0.006), and live birth rate (64.24% vs. 46.45%, adjusted p=0.004) after adjustment for confounder, including female age, female BMI, basal FSH, AMH, duration of infertility, previous IVF failures, number of prior miscarriages, IVF indication, type of infertility, endometrial thickness, number of embryos transferred, and grade/stage of embryo transfer. (**Table 2**). The baseline characteristics of the two groups were presented in **Supplementary Table 2**.

### 3.4 Pregnancy outcomes stratified by CD138 status

To further evaluate the effectiveness of the EMT-guided strategy, we compared its outcomes stratified by CD138 status. CD138-positive patients who received standard care (n= 67) and CD138-negative patients who did not undergo any intervention(n=88) were used as control groups (**Figure 1**).

#### 3.4.1 CD138-negative patients

**Figure 2:**
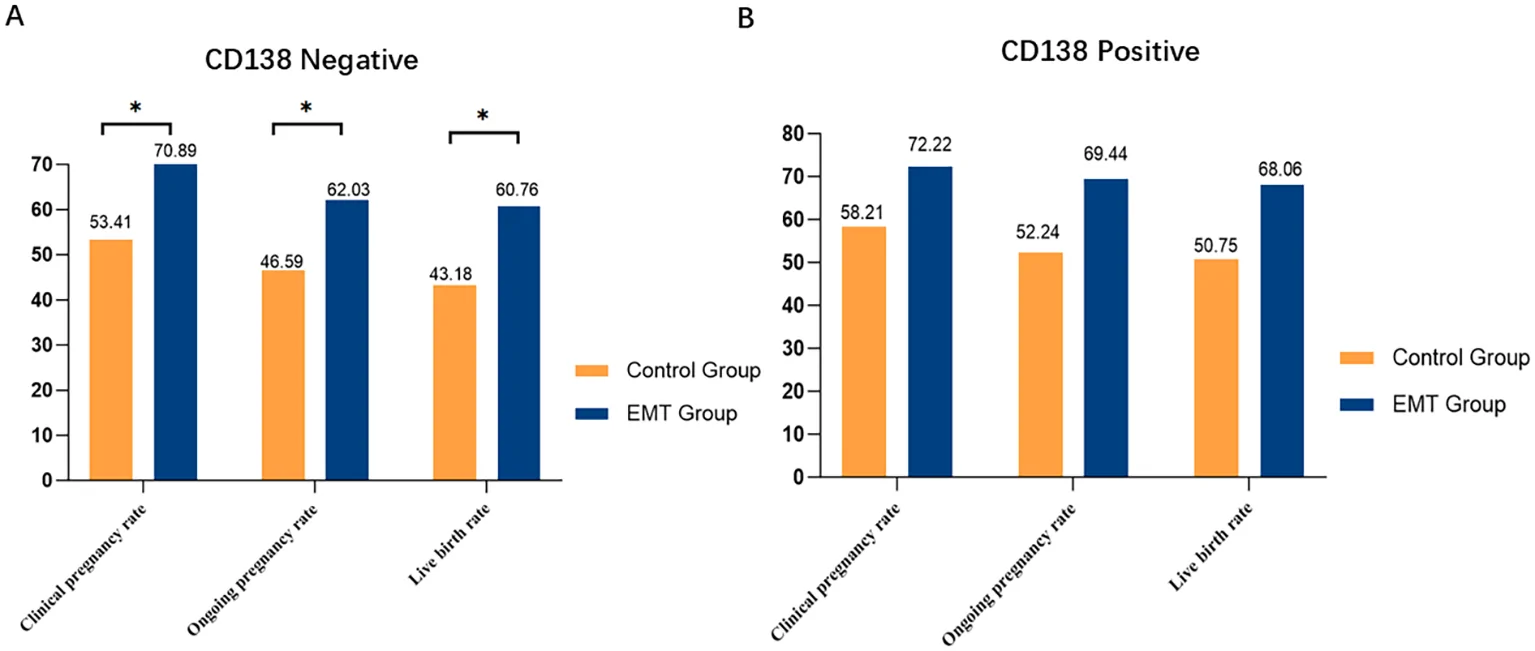
Comparison of clinical outcomes between EMT and non-EMT groups stratified by CD138 status. **(a)** Clinical Outcomes among CD138 negative patients; **(b)** Clinical Outcomes among CD138 positive patients. *p < 0.05

Among CD138-Negative Patients, the efficacy of EMT-guided personalized therapy was evaluated by comparing the EMT group (n=79) with the control group without treatment (n=88). **Supplementary Table 3** presents the baseline characteristics of the two groups. As shown in **Table 3**, after adjusting for confounders using logistic regression analysis, the clinical pregnancy rate of the EMT group was higher than that of the control group (70.89% vs 53.41%, adjusted p=0.011). The ongoing pregnancy (62.03% vs 46.59%, adjusted p=0.041) and live birth rate (60.76% vs 43.18%, adjusted p=0.030) were also higher in the EMT group (**Figure 2A**).

**Table 3:** Comparison of clinical outcomes between EMT-guided personalized therapy and no treatment in CD138-negative patients.

Outcomes	Non-EMT group	EMT group	P value	Adjusted p-value*
Clinical Pregnancy Rate	53.41% (47/88)	70.89% (56/79)	**0.020**	**0.011**
Ongoing Pregnancy Rate	46.59% (41/88)	62.03% (49/79)	**0.046**	**0.041**
Live Birth Rate	43.18% (38/88)	60.76% (48/79)	**0.023**	**0.030**

Data are presented as rates (n/N). Bold values denote a statistically significant difference (P < 0.05).

^*^ *Adjusted p-values were derived from multivariate logistic regression analysis, controlling for maternal age, female BMI, basal FSH, AMH, endometrial thickness, duration of infertility, previous IVF failures, number of prior miscarriages, IVF indication, type of infertility, number of embryos transferred, and the grade/stage of embryo transferred. IVF indication was collapsed into 5 levels (Tubal/Male/Ovulatory/Couple/Other) to avoid quasi-complete separation from sparse categories.

Clinical outcomes by EMT subgroup within the CD138-negative patients are presented in **Supplementary Table 5**. The mild dysbiosis subgroup showed the highest live birth rate. Given the limited sample size in certain subgroups, no further statistical comparison of inter-group differences was performed.

#### 3.4.2 CD138-positive patients

Among CD138-Positive Patients, **Supplementary Table 4** presents the baseline characteristics of the two groups. As shown in **Table 4**, the EMT group demonstrated higher ongoing pregnancy and live birth rates compared to the control (empirical treatment) group (69.44% vs. 52.24%, p=0.038; 68.06% vs. 50.75%, p=0.038, respectively). However, after adjusting for confounders using logistic regression analysis, the differences in ongoing pregnancy and live birth rates were no longer statistically significant (adjusted p=0.142 and p=0.118, respectively) (**Figure 2B**).

**Table 4:** Comparison of clinical outcomes between EMT-guided personalized therapy and empirical treatment in CD138-positive patients.

Outcomes	Non-EMT group	EMT group	P value	Adjusted p-value *
Clinical Pregnancy Rate	58.21% (39/67)	72.22% (52/72)	0.083	0.208
Ongoing Pregnancy Rate	52.24% (35/67)	69.44% (50/72)	**0.038**	0.142
Live Birth Rate	50.75% (34/67)	68.06% (49/72)	**0.038**	0.118

Data are presented as rates (n/N). Bold values denote a statistically significant difference (P < 0.05).

^*^ *Adjusted p-value from logistic regression analysis after controlling for: female age, female BMI, basal FSH, AMH, endometrial thickness, number of embryos transferred, duration of infertility, previous IVF failures, number of prior miscarriages, IVF indication, type of infertility, and the grade/stage of embryo transferred. IVF indication was collapsed into 5 levels (Tubal/Male/Ovulatory/Couple/Other) to avoid quasi-complete separation from sparse categories.

The clinical outcomes of each EMT subgroup within the CD138-positive patients are presented in **Supplementary Table 6**. Outcomes were similar across the four subgroups, and no further inter-group comparisons were performed due to limited sample sizes.

## 4 Discussion

In this study, we systematically evaluated the clinical application value of EMT-guided personalized treatment strategies in infertile women. Our findings indicate that the EMT-guided personalized treatment was associated with improved clinical outcomes for CD138-negative patients, whereas no statistically significant benefit was observed in CD138-positive patients after adjustment for confounders. These findings suggest that EMT may serve as a complementary diagnostic tool, particularly for patients who are negative for traditional chronic endometritis (CE) markers, although prospective studies are needed to confirm its clinical utility.

Among the 151 patients who underwent EMT testing, *lactobacilli* deficiency was highly prevalent, affecting 86.8% (131/151) of participants, whereas pathogenic bacteria were detected in only 57.0% (86/151) of patients (**Table 1**). Notably, we observed a discordance between CD138 immunohistochemistry results and EMT results: the distribution of EMT classifications did not differ significantly between CD138-negative and CD138-positive patients (p = 0.355), and the prevalence of pathogenic bacteria was comparable between the two groups (51.9% vs. 62.5%, p = 0.247) (**Table 1**). This indicates that EMT identifies microbial dysbiosis largely independent of CD138 status.

This discordance aligns with Hiratsuka et al. (2025), who reported no correlations among test-positive individuals in hysteroscopy, the endometrial CD138 test, and the endometrial microbiome test (31). The discordance results may be attributed to several factors: 1) The presence of non-bacterial causes of chronic endometritis (e.g., viral, fungal, or non-infectious inflammatory conditions); 2) limitations in the sensitivity of 16S rRNA sequencing due to low bacterial biomass or prior antibiotic treatment; 3) the possibility that the detected bacteria by 16S rRNA are non-pathogenic colonizers that do not elicit a plasma cell response. As Hiratsuka et al. (2025) suggested, the endometrial microbiome test may select a different patient population from the hysteroscopy and endometrial CD138 tests.

Following personalized treatment, the overall live birth rate in the EMT group was 64.24%. Although clinical outcomes varied across the four EMT subgroups (Normal, Mild dysbiosis, Abnormal dysbiosis, CE pathogens), statistical comparisons were not performed for subgroups with limited sample sizes (e.g., CE pathogens, n = 6) (**Table 2**). Nevertheless, the mild dysbiosis subgroup achieved the highest live birth rate (68.63%), suggesting that early intervention in patients with mild lactobacilli deficiency may be particularly beneficial.

Currently, the optimal treatment strategy for dysbiotic endometrium remains unresolved. Previous studies concluded that recovering a Lactobacillus-dominated endometrium may improve implantation outcomes (11, 32). Kadogami et al. (2020) administered a combination of oral and vaginal probiotics, or oral probiotics together with antibiotics, to patients diagnosed with non-*Lactobacillus*-dominated microbiota (NLDM) in the endometrium (33). Our study implemented a more refined, phenotype-guided approach. Patients were stratified into four distinct subgroups according to EMT profiles, with each subgroup receiving a differentiated treatment protocol. This strategy achieved encouraging live birth rates, with the mild dysbiosis subgroup achieving 68.63%—suggesting that early intervention in patients with *Lactobacillus* deficiency may be particularly beneficial. The improvement results align with growing evidence that a *Lactobacillus*-dominated intrauterine microbiome is associated with better reproductive success (12, 13). *Lactobacillus* species are known to produce lactic acid, which maintains a low pH environment, inhibits the growth of pathogenic bacteria, and modulates local immune responses, thereby creating a favorable milieu for embryo implantation (34, 35).

In CD138-negative patients, EMT-guided personalized therapy showed a significant relationship with higher clinical pregnancy and live birth rates than the control group (No treatment), even after adjustment for confounders. The diagnostic criteria for CD138 positivity remain controversial, with varying thresholds reported across studies (36). Consequently, some patients with true endometrial pathology may be misclassified as CD138-negative by conventional histology. Our findings indicate that EMT can identify a subset of these “missed” patients who may benefit from microbiome-directed therapy, highlighting the complementary role of molecular diagnostics in comprehensive endometrial assessment.

However, the improved outcomes in this group may not solely reflect the “rescue” of missed CE cases. An alternative interpretation of the benefit is that CD138-negative patients with EMT-detected dysbiosis represent a distinct entity—endometrial dysbiosis without established chronic inflammation. In this scenario, the endometrial stroma remains functionally preserved, and these patients may exhibit greater responsiveness to antibiotic or probiotic intervention than those with histologically confirmed CE, in whom chronic inflammation leads to stromal remodeling, immune microenvironment dysregulation, and impaired endometrial receptivity that may persist beyond microbiological eradication. The present study cannot distinguish between these two hypotheses, as histological, immunological, and microbiological data were not co-assessed. Prospective studies integrating these assessments will be required to clarify the underlying mechanism.

In contrast, among CD138-positive patients, EMT-guided therapy showed no significantly improvement for ongoing pregnancy (69.44% vs. 52.24%, adjusted p=0.142) and live birth rates (68.06% vs. 50.75%, adjusted p=0.118) compared to empirical treatment after adjustment for confounders. This indicates that empirical antibiotic therapy itself may be effective for a majority of CE cases, as previous studies report (8, 37), leaving a smaller margin for further improvement through refined microbiota targeting. However, a consistent trend toward better outcomes was observed; larger prospective studies are needed to confirm whether this personalized approach provides a meaningful benefit over standard empirical treatment.

More importantly, our EMT-based stratification demonstrates that not all such patients require antibiotic intervention. Specifically, among CD138-positive individuals with either normal endometrial microbiota or only mild dysbiosis—comprising 36.1% (26/72) of this cohort—favorable pregnancy outcomes were observed with probiotics alone or even without treatment (**Supplementary Table 6**), illustrating the critical role of EMT in preventing antibiotic overtreatment and enabling safer, more rational therapy.

Chen et al. reported that the composition of endometrial microorganisms in CE and non-CE patients was significantly different (38). The use of 16S rRNA sequencing to guide personalized treatment enables the precise identification of microbial taxa and the assessment of microbial diversity, allowing clinicians to tailor interventions to the unique microbiome profile of each patient [8]. While previous studies have explored the relationship between the intrauterine microbiome and IVF outcomes, few have investigated the impact of microbiome-based interventions on reproductive success (16). Our findings support an optimized two-step diagnostic model: initial histological assessment (CD138), followed by EMT analysis in CD138-negative cases to guide precision therapy. Such a strategy aligns with the principles of precision medicine and could optimize resource allocation in clinical practice.

Several limitations should be acknowledged. First, the retrospective, single-center design of the study may introduce the potential for selection bias and limit the generalizability of the findings. Although we adjusted for available covariates, residual confounding cannot be excluded. Second, the sample size of certain subgroups, particularly the CE pathogens group (n = 6), was too small to permit meaningful statistical comparisons or robust inferences. Third, post-treatment reassessment of endometrial status was not performed in either group before embryo transfer, as repeat biopsy is not routine at our center. Thus, we cannot rule out the possibility that persistent infection or inflammation in some patients across both groups may have influenced the observed outcomes. Fourth, the mechanisms by which specific microbial taxa influence reproductive outcomes remain incompletely understood and warrant further investigation. Large-scale, prospective randomized controlled trials are warranted to conclusively validate these findings and refine the specific treatment protocols for each microbiota profile.

## 5 Conclusions

Our findings provide preliminary evidence that an EMT-guided personalized therapy strategy appeared to be linked to improved live birth rates in CD138-negative infertility patients, identifying a group that may benefit from EMT intervention. For CD138-positive patients, EMT-guided therapy showed no statistically significant superiority over empirical antibiotic treatment, although numerically higher rates were observed. These findings support a role for EMT as a complementary diagnostic tool in comprehensive endometrial assessment, particularly in CD138-negative patients with clinical indications. Prospective randomized trials are warranted to validate these observations and establish optimal treatment algorithms for different patient subgroups.

## Data Availability

The raw data supporting the conclusions of this article will be made available by the authors, without undue reservation.
