# Supplementary material for: Efficacy of endometrial microbiota testing-guided personalized therapy in infertile women stratified by CD138 status: a retrospective cohort study
Source: Front Endocrinol (Lausanne). 2026 Aug 26;17:1849095. doi: 10.3389/fendo.2026.1849095 (PMC13553447; doi:10.3389/fendo.2026.1849095)
Supplement: Supplementary file 1 [file Table1.docx]

Supplementary Material

# 1 Supplementary Tables

**Supplementary Table 1. Recommended Antimicrobial Therapy for Pathogens***

| **Pathogen** | **First-line Regimen** | **Alternative Regimen** |
| --- | --- | --- |
| ***Escherichia* spp.** | Oral cephalosporins (cefdinir, cefotaxime)  Aminoglycosides (gentamicin, amikacin) | Fluoroquinolones (e.g., ofloxacin, ciprofloxacin)  Parenteral cephalosporins (e.g., ceftazidime-avibactam) |
| ***Klebsiella* spp.** | Piperacillin-tazobactam  Ceftriaxone | Amoxicillin-clavulanate, Ampicillin-sulbactam  Parenteral cephalosporins, Fluoroquinolones  Carbapenems (doripenem, ertapenem) |
| ***Shigella* spp.** | Ciprofloxacin | Azithromycin |
| ***Neisseria* spp.** | Ceftriaxone | Azithromycin (high-dose) |
| ***Enterococcus* spp.** | Penicillin G, Ampicillin, Amoxicillin  Vancomycin, Teicoplanin, Linezolid | Amoxicillin-clavulanate, Ampicillin-sulbactam  Piperacillin-tazobactam, Imipenem-cilastatin  Fluoroquinolones, Tigecycline, Nitrofurantoin, etc. |
| ***Staphylococcus* spp.** | Oxacillin, Nafcillin, Cloxacillin, Flucloxacillin, Dicloxacillin | Parenteral cephalosporins  Vancomycin, Teicoplanin, Telavancin |
| ***Streptococcus* spp.** | Penicillin G  Cephalosporins | Clindamycin, Vancomycin, Tigecycline  Oral/Parenteral cephalosporins |
| ***Chlamydia*** | Doxycycline; Azithromycin | Erythromycin, Ofloxacin, Levofloxacin |
| ***Mycoplasma*** |  |  |
| ***Ureaplasma*** |  |  |
| ***M. tuberculosis*** | Extrapulmonary TB:  Intensive (2 mo): INH + RFP + PZA + EMB, qd.  Continuation: INH + RFP. | / |

* Reference

[1] D.N. Gilbert, et al. The Sanford guide to antimicrobial therapy 2024 (54th Edition). 2024.,

[2] CHINET China Antimicrobial Resistance Surveillance Network, http://www.chinets.com/

**Supplementary Table 2. Baseline Clinical Characteristics of All Patients in the Non-EMT and EMT Groups**

| **Variables** | **Non-EMT Group**  **(n = 155)** | **EMT Group**  **(n = 151)** | **P value** |
| --- | --- | --- | --- |
| Female age (Mean ± SD) | 31.63 ± 3.79 | 31.83 ± 3.49 | 0.628 |
| Male age (Mean ± SD) | 33.85 ± 4.32 | 33.56 ± 4.18 | 0.558 |
| Female BMI (Mean ± SD) | 22.58 ± 3.48 | 22.97 ± 3.58 | 0.336 |
| Endometrium thickness, mm, Mean ± SD | 9.75 ± 1.90 | 10.08 ± 1.68 | 0.101 |
| AMH, Median (Q₁, Q₃) | 3.51 (2.06, 5.48) | 3.88 (2.33, 5.68) | 0.449 |
| Basal FSH, Median (Q₁, Q₃) | 6.09 (5.25, 7.44) | 6.24 (5.38, 7.19) | 0.965 |
| Duration of infertility, Median (Q₁, Q₃) | 2.00 (2.00, 5.00) | 3.00 (1.00, 5.00) | 0.910 |
| No. of prior miscarriages, Median (Q₁, Q₃) | 0.00 (0.00, 1.00) | 0.00 (0.00, 1.00) | 0.653 |
| No. of prior implantation failures, n (%) |  |  | **0.005** |
| 0 | 139 (89.68) | 114 (76.00) |  |
| 1 | 13 (8.39) | 25 (16.67) |  |
| ≥2 | 3 (1.94) | 11 (7.33) |  |
| IVF indication, n(%) |  |  | 0.937 |
| Tubal factor | 106 (68.39) | 105 (69.54) |  |
| Male factor | 17 (10.97) | 12 (7.95) |  |
| Couple factor | 10 (6.45) | 11 (7.28) |  |
| Endometriosis | 4 (2.58) | 2 (1.32) |  |
| Other | 7 (4.52) | 9 (5.96) |  |
| Ovulatory dysfunction | 6 (3.87) | 7 (4.64) |  |
| Unexplained | 5 (3.23) | 5 (3.31) |  |
| Type of infertility, n(%) |  |  | 0.217 |
| Primary infertility | 64 (41.29) | 52 (34.44) |  |
| Secondary infertility | 91 (58.71) | 99 (65.56) |  |
| Method of fertilization, n(%) |  |  | 0.838 |
| IVF | 90 (58.06) | 81 (54.00) |  |
| ICSI | 62 (40.00) | 66 (44.00) |  |
| IVF+ICSI | 3 (1.94) | 3 (2.00) |  |
| Number of embryos transferred, n(%) |  |  | 0.257 |
| 1 | 46 (29.68) | 54 (35.76) |  |
| 2 | 109 (70.32) | 97 (64.24) |  |
| Stage/grade of embryo transferred, n (%) |  |  | **0.003** |
| Blastocyst-Good (AA/BA/AB) | 4 (2.65) | 6 (3.87) |  |
| Blastocyst-Fair (BB) | 48 (31.79) | 23 (14.84) |  |
| Blastocyst-Poor (BC/CB) | 59 (39.07) | 63 (40.65) |  |
| Cleavage-Good* | 34 (22.52) | 59 (38.06) |  |
| Cleavage-Medium | 6 (3.97) | 4 (2.58) |  |

Values are reported as median and interquartile range, or as number and percentage. Abbreviations: BMI, body mass index; AMH, anti-Mullerian hormone; FSH, follicle-stimulating hormone; EMT: endometrial microbiota test.

*High-quality cleavage embryo = 7–9 cells + Grade I/II.

**Supplemental Table 3 Baseline clinical characteristics of the CD138-negative patients in the Non-EMT group and EMT group**

| **Variables** | **Non-EMT Group  (n = 88)** | **EMT group  (n = 79)** | **P value** |
| --- | --- | --- | --- |
| Female age (Mean ± SD) | 31.48 ± 3.99 | 31.82 ± 3.64 | 0.561 |
| Male age (Mean ± SD) | 33.55 ± 3.81 | 33.53 ± 3.81 | 0.973 |
| Female BMI (Mean ± SD) | 22.87 ± 3.51 | 23.12 ± 3.68 | 0.660 |
| Endometrium thickness, mm, Mean ± SD | 9.81 ± 1.75 | 10.05 ± 1.59 | 0.355 |
| AMH, Median (Q₁, Q₃) | 3.47 (2.16, 5.68) | 3.89 (2.53, 5.77) | 0.565 |
| Basal FSH, Median (Q₁, Q₃) | 6.37 (5.62, 7.47) | 6.26 (5.35, 7.19) | 0.247 |
| Duration of infertility, Median (Q₁, Q₃) | 2.00 (2.00, 5.00) | 3.00 (1.00, 5.00) | 0.478 |
| No. of prior miscarriages, Median (Q₁, Q₃) | 0.00 (0.00, 1.00) | 1.00 (0.00, 1.00) | 0.740 |
| No. of prior implantation failures, n (%) |  |  | 0.008 |
| 0 | 78 (88.64) | 54 (69.23) |  |
| 1 | 7 (7.95) | 17 (21.79) |  |
| ≥2 | 3 (3.41) | 7 (8.97) |  |
| IVF indication, n (%) |  |  | 0.794 |
| Tubal factor | 63 (71.59) | 54 (68.35) |  |
| Male factor | 8 (9.09) | 7 (8.86) |  |
| Couple factor | 9 (10.23) | 10 (12.66) |  |
| Endometriosis | 1 (1.14) | 1 (1.27) |  |
| Other | 1 (1.14) | 2 (2.53) |  |
| Ovulatory dysfunction | 6 (6.82) | 3 (3.80) |  |
| Unexplained | 0 (0.00) | 2 (2.53) |  |
| Type of infertility, n (%) |  |  | 0.536 |
| Primary infertility | 33 (37.50) | 26 (32.91) |  |
| Secondary infertility | 55 (62.50) | 53 (67.09) |  |
| Method of fertilization, n (%) |  |  | 0.434 |
| IVF | 54 (61.36) | 41 (51.90) |  |
| ICSI | 32 (36.36) | 36 (45.57) |  |
| IVF+ICSI | 2 (2.27) | 2 (2.53) |  |
| No. of embryos transferred, n (%) |  |  | 0.252 |
| 1 | 25 (28.41) | 29 (36.71) |  |
| 2 | 63 (71.59) | 50 (63.29) |  |
| Stage/grade of embryo transferred, n (%) |  |  | 0.032 |
| Blastocyst-Good (AA/BA/AB) | 4 (4.55) | 2 (2.53) |  |
| Blastocyst-Fair (BB) | 14 (15.91) | 25 (31.65) |  |
| Blastocyst-Poor (BC/CB) | 34 (38.64) | 34 (43.04) |  |
| Cleavage-Good* | 33 (37.50) | 15 (18.99) |  |
| Cleavage-Medium | 3 (3.41) | 3 (3.80) |  |

Values are median and interquartile range, or number and percentage. Abbreviations: BMI, body mass index; AMH, anti-Mullerian hormone; FSH, follicle-stimulating hormone; EMT: endometrial microbiota test.

*High-quality cleavage embryo = 7–9 cells + Grade I/II.

**Supplemental Table 4 Baseline clinical characteristics of the CD138-positive patients in the non-EMT group and EMT group**

| **Variables** | **Non-EMT Group  (n = 67)** | **EMT group  (n = 72)** | **P value** |
| --- | --- | --- | --- |
| Female age (Mean ± SD) | 31.84 ± 3.53 | 31.85 ± 3.35 | 0.984 |
| Male age (Mean ± SD) | 34.24 ± 4.91 | 33.60 ± 4.56 | 0.426 |
| Female BMI (Mean ± SD) | 22.20 ± 3.43 | 22.81 ± 3.49 | 0.302 |
| Endometrium thickness, mm, Mean ± SD | 9.67 ± 2.08 | 10.13 ± 1.78 | 0.169 |
| AMH, Median (Q₁, Q₃) | 3.54 (2.07, 5.25) | 3.86 (2.18, 5.54) | 0.579 |
| Basal FSH, Median (Q₁, Q₃) | 5.85 (4.85, 7.00) | 6.21 (5.46, 7.20) | 0.151 |
| Duration of infertility, Median (Q₁, Q₃) | 3.00 (1.00, 4.50) | 3.00 (1.00, 4.00) | 0.547 |
| No of prior miscarriages, Median (Q₁, Q₃) | 0.00 (0.00, 1.00) | 0.00 (0.00, 1.00) | 0.352 |
| No. of prior implantation failures, n (%) |  |  | 0.169 |
| 0 | 61 (91.04) | 60 (83.33) |  |
| 1 | 6 (8.96) | 8 (11.11) |  |
| ≥2 | 0 (0.00) | 4 (5.56) |  |
| IVF indication, n (%) |  |  | 0.295 |
| Tubal factor | 43 (64.18) | 51 (70.83) |  |
| Male factor | 9 (13.43) | 5 (6.94) |  |
| Couple factor | 1 (1.49) | 1 (1.39) |  |
| Endometriosis | 3 (4.48) | 1 (1.39) |  |
| Other | 6 (8.96) | 7 (9.72) |  |
| Ovulatory dysfunction | 0 (0.00) | 4 (5.56) |  |
| Unexplained | 5 (7.46) | 3 (4.17) |  |
| Type of infertility, n (%) |  |  | 0.224 |
| Primary infertility | 31 (46.27) | 26 (36.11) |  |
| Secondary infertility | 36 (53.73) | 46 (63.89) |  |
| Method of fertilization, n (%) |  |  | 0.931 |
| IVF | 36 (53.73) | 40 (56.34) |  |
| ICSI | 30 (44.78) | 30 (42.25) |  |
| IVF+ICSI | 1 (1.49) | 1 (1.41) |  |
| No. of embryos transferred, n (%) |  |  | 0.672 |
| 1 | 21 (31.34) | 25 (34.72) |  |
| 2 | 46 (68.66) | 47 (65.28) |  |
| Stage/grade of embryo transferred, n (%) |  |  | 0.061 |
| Blastocyst-Good (AA/BA/AB) | 2 (2.99) | 2 (2.78) |  |
| Blastocyst-Fair (BB) | 9 (13.43) | 23 (31.94) |  |
| Blastocyst-Poor (BC/CB) | 29 (43.28) | 25 (34.72) |  |
| Cleavage-Good* | 26 (38.81) | 19 (26.39) |  |
| Cleavage-Medium | 1 (1.49) | 3 (4.17) |  |

Values are median and interquartile range, or number and percentage. Abbreviations: BMI, body mass index; AMH, anti-Mullerian hormone; FSH, follicle-stimulating hormone;EMT: endometrial microbiota test

*High-quality cleavage embryo = 7–9 cells + Grade I/II.

**Supplemental Table 5 Clinical Outcomes of the CD138-Negative Subgroup Stratified by EMT result.**

| **Outcomes** | **Abnormal (n = 38)** | **Mild (n = 30)** | **CE_patho (n = 2)** | **Normal (n = 9)** |
| --- | --- | --- | --- | --- |
| Clinical Pregnancy, n(%) | 24 (63.16) | 25 (83.33) | 0 (0.00) | 7 (77.78) |
| Ongoing Pregnancy, n(%) | 21 (55.26) | 22 (73.33) | 0 (0.00) | 6 (66.67) |
| Live Birth, n(%) | 21 (55.26) | 21 (70.00) | 0 (0.00) | 6 (66.67) |

**Supplemental Table 6 Clinical Outcomes of the CD138-positive Subgroup Stratified by EMT result.**

| **Outcomes** | **Abnormal (n = 42)** | **Mild (n = 21)** | **CE_patho (n = 4)** | **Normal (n = 5)** |
| --- | --- | --- | --- | --- |
| Clinical Pregnancy, n(%) | 30 (71.43) | 14 (66.67) | 3 (75.00) | 5 (100.00) |
| Ongoing Pregnancy, n(%) | 28 (66.67) | 14 (66.67) | 3 (75.00) | 5 (100.00) |
| Live Birth, n(%) | 28 (66.67) | 14 (66.67) | 3 (75.00) | 4 (80.00) |
